# The First Case Report of Preschool-Onset SS/SLE Coexisting With NMOSD of Chinese Origin

**DOI:** 10.3389/fimmu.2022.887041

**Published:** 2022-05-02

**Authors:** Liqun Liu, Li Tang, Lu Zhang, Xingfang Li, Peng Huang, Jie Xiong, Yangyang Xiao, Lingjuan Liu

**Affiliations:** ^1^ Department of Pediatrics, The Second Xiangya Hospital of Central South University, Changsha, China; ^2^ Department of Pediatric Neurology, Children’s Medical Center, The Second Xiangya Hospital of Central South University, Changsha, China

**Keywords:** neuromyelitis optica spectrum disorder, systemic lupus erythematosus, Sjogren’s syndrome, AQP-4, child SS/SLE overlap syndrome with NMOSD

## Abstract

Systemic lupus erythematosus (SLE) is an autoimmune connective tissue disease (CTD), the main features of which are multiple serum autoantibodies and extensive involvement of multiple systems. The onset age of patients varies from childhood to middle age, with nearly 1/5 in childhood. Sjogren’s syndrome (SS) is also an autoimmune disease characterized by high-degree lymphocytic infiltration of exocrine glands, usually occurring in middle-aged and older women, and rarely in childhood. Neuromyelitis optica spectrum disorder (NMOSD) is an immune-mediated inflammatory demyelinating disease of the central nervous system (CNS) mainly involving the optic nerve and spinal cord. The coexistence of NMOSD and SLE and/or SS is well recognized by both neurologists and rheumatologists, but cases in children have been rarely reported. In this paper, we reported a case of a girl with onset at age 5 clinically featured by recurrent parotid gland enlargement, pancytopenia, hypocomplementemia, multiple positive serum antibodies, and cirrhosis. She was initially diagnosed with SS/SLE overlap syndrome at age 5. Four years later, the patient suffered a sudden vision loss and was examined to have positive AQP4 antibodies in serum and cerebrospinal fluid (CSF), and long segmental spinal swelling, in line with the diagnostic criteria for NMOSD. Up to now, the current patient is of the youngest onset age to develop SS/SLE coexisting with NMOSD, also with cirrhosis. It is important for clinicians to be aware of the possibility of CTDs coexisting with NMOSD in children, especially in those with positive anti-multiple autoantibodies, and to decrease the rate of missed diagnosis.

## Introduction

SLE is a diffuse connective tissue disease mediated by autoimmunity, which is characterized by the presence of multiple serum autoantibodies and multi-system involvement. Neuromyelitis optica spectrum disorder (NMOSD) is an autoimmune disease of the central nervous system that is associated with serum aquaporin-4 antibodies (AQP4-IgG) directly against the AQP4 channels on the foot processes of astrocytes (1), clinically with serious optic neuritis (ON), longitudinally extensive transverse myelitis (LETM), and intractable nausea, vomiting, and hiccups induced by area postrema syndrome (APS) as the main manifestations (2). AQP4-IgG is the amplification of inflammation that mediates the damage of the blood–brain barrier (BBB), astrocyte injury, subsequent demyelination, and finally the occurrence and development of NMOSD (3). Therefore, AQP4-IgG is considered a pathogenic marker of NMOSD, and the presence of AQP4-IgG unequivocally distinguishes NMOSD from multiple sclerosis (MS) (4). NMOSD is more likely to coexist with SLE (5), and with the probability of 1/5,000,000 (6). The earliest description of SLE with NMOSD was reported in 1976 in a young woman who initially presented with enuresis and urinary incontinence but developed retrobulbar optic neuritis at the terminal stage of the disease, meeting the diagnostic criteria for SLE. Similar cases have been reported in greater numbers, but cases in children are still rare.

Sjogren’s syndrome (SS) is an autoimmune systemic disease, characterized by the presence of anti-SSA/Ro antibodies in serum, dry eyes, and a dry mouth (7). More than 90% of patients with SS are women, mostly middle-aged women (8), with rare cases reported in children. Recently, patients with NMOSD (AQP4-IgG-positive) coexisting with SS have been reported more frequently than before (9), and different clinical manifestations might be presented based on distinct pathomechanisms (10). In an AQP4-IgG seropositive NMOSD cohort, autoimmune comorbidities were reported in 35.1% of patients, and systemic autoantibodies [e.g., anti-SSA and anti-SSB antibodies] and anti-ANA antibodies were reported in 51.4% of patients (11). Therefore, some clinicians believe that NMOSD is a central nervous system complication of SS, whereas recent studies have shown that NMOSD and SS can coexist as two different diseases.

This paper reported a case of a girl with recurrent parotid gland enlargement and pancytopenia as initial symptoms at the age of 5. During the course of the disease, she also suffered from liver dysfunction and jaundice, and imaging diagnosed liver cirrhosis. Four years after the onset, vision loss and positive AQP4-IgG appeared in serum and CSF. The patient was finally diagnosed as the coexistence of SS/SLE with NMOSD. Moreover, the relevant literature review was performed.

## Case Presentation

**Figure 1:**
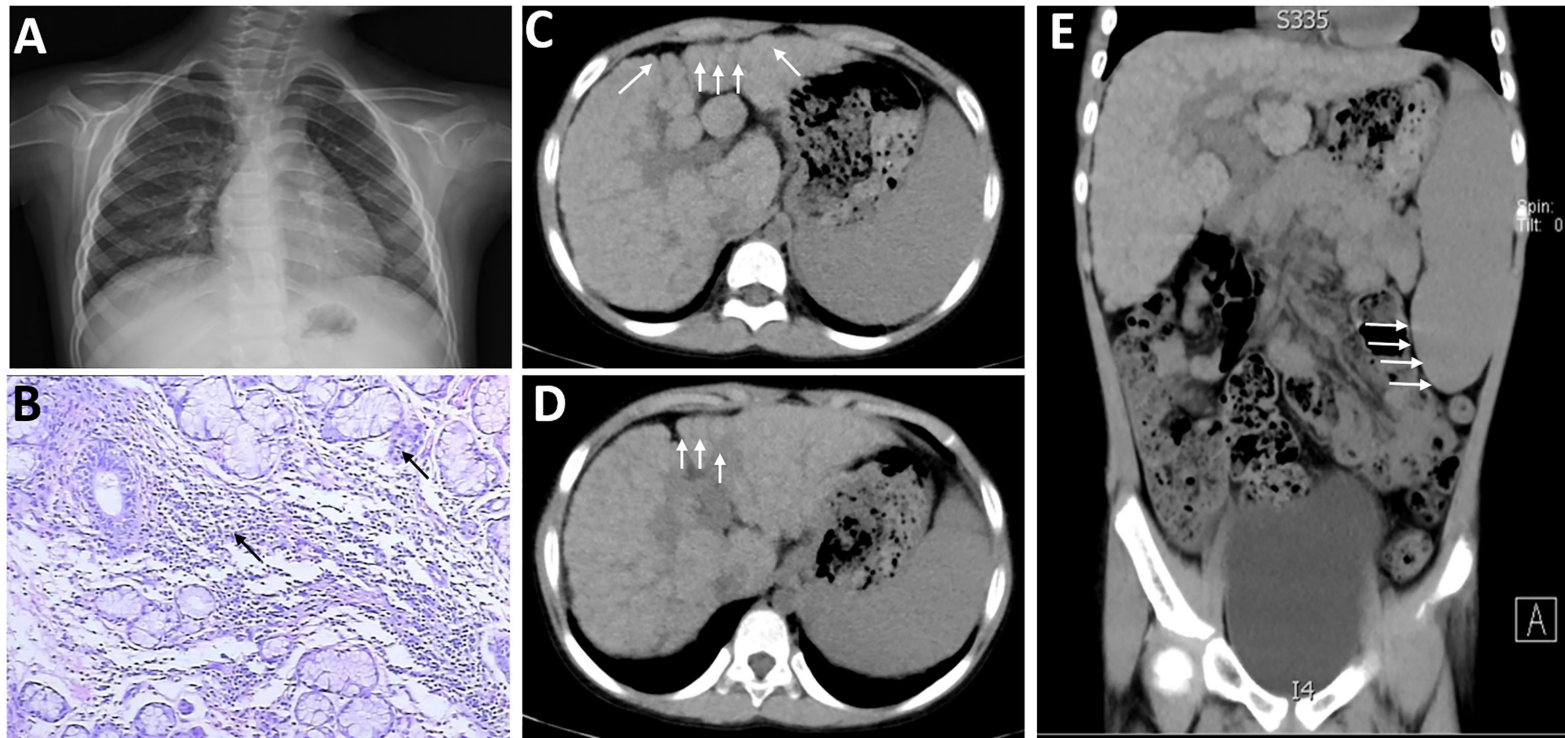
Imaging features of the patient with both systemic lupus erythematosus (SLE) and Sjogren’s syndrome (SS) when she was 5 years old. The textures of both lungs were increased and blurred, and there were small patchy shadows **(A)**. There were 2 small foci of lymphocyte invasion in salivary gland tissue microscopically (>50/focus) after labial gland biopsy **(B)**. **(C–E)** The volume of the liver was reduced, and each lobe was out of proportion, especially the right lobe. The left and caudal lobes of the liver were enlarged and the fissure was widened. The surface of the liver was not smooth, small nodular protrusions could be seen (shown by arrows), and the density of liver parenchyma was not uniform. The spleen was markedly enlarged and about 6 cm in thickness (shown by arrows).

The current patient developed parotid gland enlargement and epistaxis, occasional fever, dry skin all over the body, multiple dental caries, and no skin rash or joint pain in January 2014 (when she was 5 years old). **Table 1** is a summary of the patient’s clinical course and treatments. She was then treated with cefotaxime for anti-infection, atomolan for protecting the liver and lowering transaminase, and methylprednisolone for immune-suppressive therapy. Two months later, lower leucocyte neutrophil, hemoglobin, and platelet amounts were detected through routine blood tests, with a recurrent parotid gland enlargement. The previous condition of the patient was poor, and she suffered from recurrent respiratory tract infections before the age of 5. Physical examination on her first admission detected that the whole-body skin was dry and cracked, more significantly in both low extremities, and with a little desquamation. There was palpable swelling in the left parotid region and without tenderness. Cardiopulmonary examination showed no abnormalities. Her abdomen was flat and soft, the liver was palpable 2 cm below the costal margin, soft in texture, and with a sharp edge. The spleen was enlarged, the A–B line was 7 cm, and there was no tenderness. Pathological signs of the nervous system were negative. Among auxiliary examinations, liver function tests showed ALT 107.6 U/L, AST 174.5 U/L, total urinary protein in 24 h was 179.50 mg/L, and erythrocyte sedimentation rate (ESR) was 41 mm/h. The regular tests of renal function, coagulation, blood lipid, copper protein, ferritin, thyroid function, gastrointestinal barium meal, chest radiograph (**Figure 1A**), and funduscopic examination all showed normal results. Rheumatoid factor, anti-ANA, anti-SSA/Ro, anti-SSB, and anti-cardiolipin antibodies were all strongly positive, while anti-double-stranded DNA (anti-dsDNA) and anti-Smith (anti-Sm) antibodies were both negative. Complement C3 and C4 reduced to 0.17 g/L and 0.12 g/L, respectively. Hyaluronic acid increased to 248.0 μg/L, and the levels of type IV collagen, laminin, and N-terminal peptide of type III procollagen were normal. Anti-histiocytic, anti-liver and kidney microsomal, anti-nuclear extract, anti-mitochondrial, and anti-hepatocellular solute antigen type 1 antibodies and anti-soluble liver antigen were all negative. Additionally, there were 2 small foci of lymphocyte invasion in salivary gland tissue microscopically (>50/focus) after labial gland biopsy (**Figure 1B**). Color ultrasound showed bilateral parotid gland swelling, chronic inflammation of the liver, and an enlarged spleen. Abdominal CT scan suggested cirrhosis and also splenomegaly (**Figures 1C–E**). Finally, she was diagnosed with SS/SLE and received prednisone and mycophenolate mofetil to treat immunosuppression. Routine blood tests showed pancytopenia repeatedly, with the lowest hemameba of 1.07×10^9^/L, platelets of 10×10^9^/L, and hemoglobins of 93 g/L. Regrettably, the patient did not take medicine regularly since September 2016. Six months later, she coughed again with sputum, and slight jaundice and icteric sclera appeared, accompanied by massive epistaxis. Subsequently, she was treated with high-dose intravenous methylprednisolone pulse therapy for 3 days followed by oral prednisone, gamma globulin hydroxychloroquine, and rituximab to inhibit immunoreactions. From November 29, 2016, to April 6, 2018, rituximab was successively administered 4 times; the patient was in a stable condition, but still with pancytopenia, cirrhosis, and continuous positive anti-SSA and anti-SSB antibodies.

**Table 1:** Timeline of events.

Date	Symptoms	Lab findings	AQP4-ab Stutus	Imageological findings	Therapy adminstered
Jan 2014	Parotid gland enlargement, oral cavity multiple caries, epistaxis, fever	ALT and AST were both increased, anti-SSA, anti-SSB, anti-ANA and anti-cardiolipin antibodies were positive, complement C3 and C4 were decreased significantly.	**/**	**/**	Methylprednisolone for immune-suppressive therapy, cefotaxime for anti-infection, and atomoran to protect liver and lowering transaminase.
Apr 2014	/	Pancytopenia, EB-DNA positive.	/	/	Ganciclovir for antiviral, prednisone and mycophenolate mofetil to treat immunosuppression.
Sep 2014	Recurrent parotid gland enlargement and epistaxis	EB-DNA positive.			
Dec 2014	Recurrent parotid gland enlargement and epistaxis	Pancytopenia, anti-SSA, anti-SSB, anti-ANA and anti-cardiolipin antibodies were positive,hypocomplementemia.	/	/	Prednisone and mycophenolate mofetil to treat immunosuppression.
Nov 2016	Recurrent parotid gland enlargement	ALT107.6u/l,AST174.5u/l,Pancytopenia,anti-SSA, anti-SSB, anti-ANA and anti-cardiolipin antibodies were positive, hypocomplementemia.	/	/	Methylprednisolone for immune-suppressive therapy, cefotaxime for anti-infection, atomoran and compound glycyrrhizin to protect liver and lowering transaminase.
Nov 2016 To Apr 2018	Respiratory tract infection,epistaxis and jaundice	ALT74.8u/l,AST186.6u/l,TBA126.7umol/l, anti-SSA, anti-SSB antibodies and rheumatoid factor were positive, hypocomplementemia.	/	Abdominal CT scan suggested cirrhosis and also splenomegaly	High-dose intravenous methylprednisolone pulse therapy for 3 days followed by oral prednisone, gamma globulin hydroxychloroquine and rituximab in inhibition of immunoreactions.
Oct 2018	A sudden progressive decline in vision, with dry eyes, lack of tears when crying, but no eye pain	Anti-ANA, anti-C1q and anti-SSA/Ro antibodies were strongly positive, also with hypocomplementemia.	AQP4-IgG in CSF and serum were with titers of 1:10 and 1:3200, respectively	Lung CT scan showed interstitial lesions and infections in both lungs, abdominal CT scan suggested cirrhosis and also splenomegaly	High-dose intravenous methylprednisolone for 3 days followed by oral prednisone, gamma globulin, and rituximab (totally 3 times) to suppress the immune reaction again.
Api 2019	/	/	/	/	Oral prednisone gradually decreases, and the dose of mycophenolate mofetil maintained.
Oct 2019	Cough aggravation and appear shortness of breath, repeated epistaxis	ALT and AST were normal, thrombopenia and hypocomplementemia.	/	/	Received continuous positive airway pressure (CPAP) to assist respiration due to respiratory failure. rituximab (totally 2 times) to suppress the immune reaction again, mycophenolate mofetil and tacrolimus to suppress the immune reaction, cefixime and cotrimoxazole for anti-infection.
Since 2019	Respiratory tract infection and epistaxis	Hypoleucemia and thrombopenia.	/	/	Oral prednisone, mycophenolate mofetil and tacrolimus to suppress the immune reaction.

**Figure 2:**
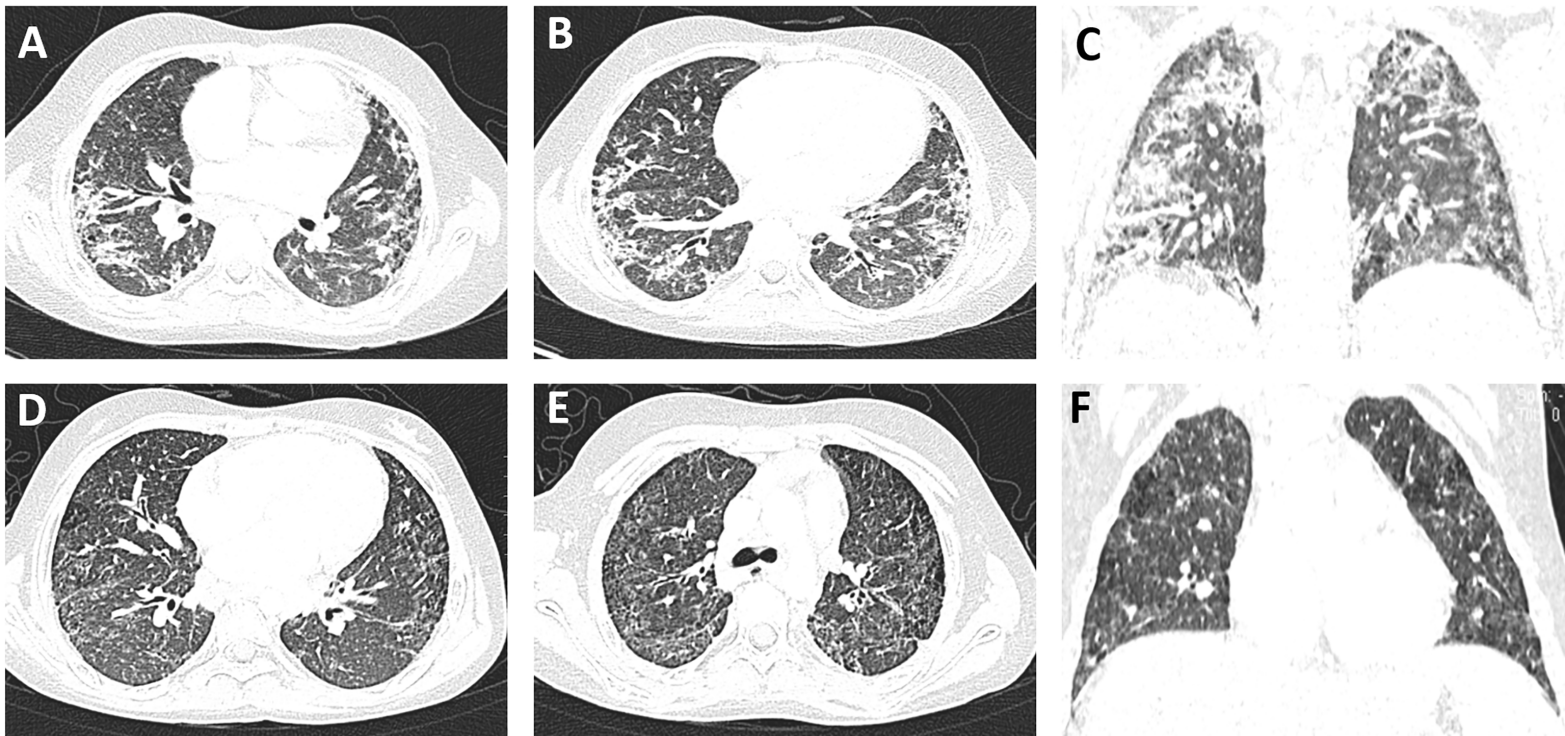
Pulmonary imaging changes of the patient with systemic lupus erythematosus (SLE)/Sjogren’s syndrome (SS) and NMOSD. **(A–C)** In December 2019, the texture of both lungs was slightly increased and disorganized, and the diffuse distribution of high-density shadows in both lungs was rod-shaped and flaky, some of which were grid-shaped with blurred edges. The cystic transparent shadows could be seen in both upper lungs, no abnormality was observed in the hilar area of both lungs, and multiple enlarged lymph nodes could be seen in the mediastinum. After the anti-infection treatment with meropenem and teicoranin, the exudation of both lungs was significantly reduced 1 month later **(D–F)**, while significant lung interstitial lesions still existed.

**Figure 3:**
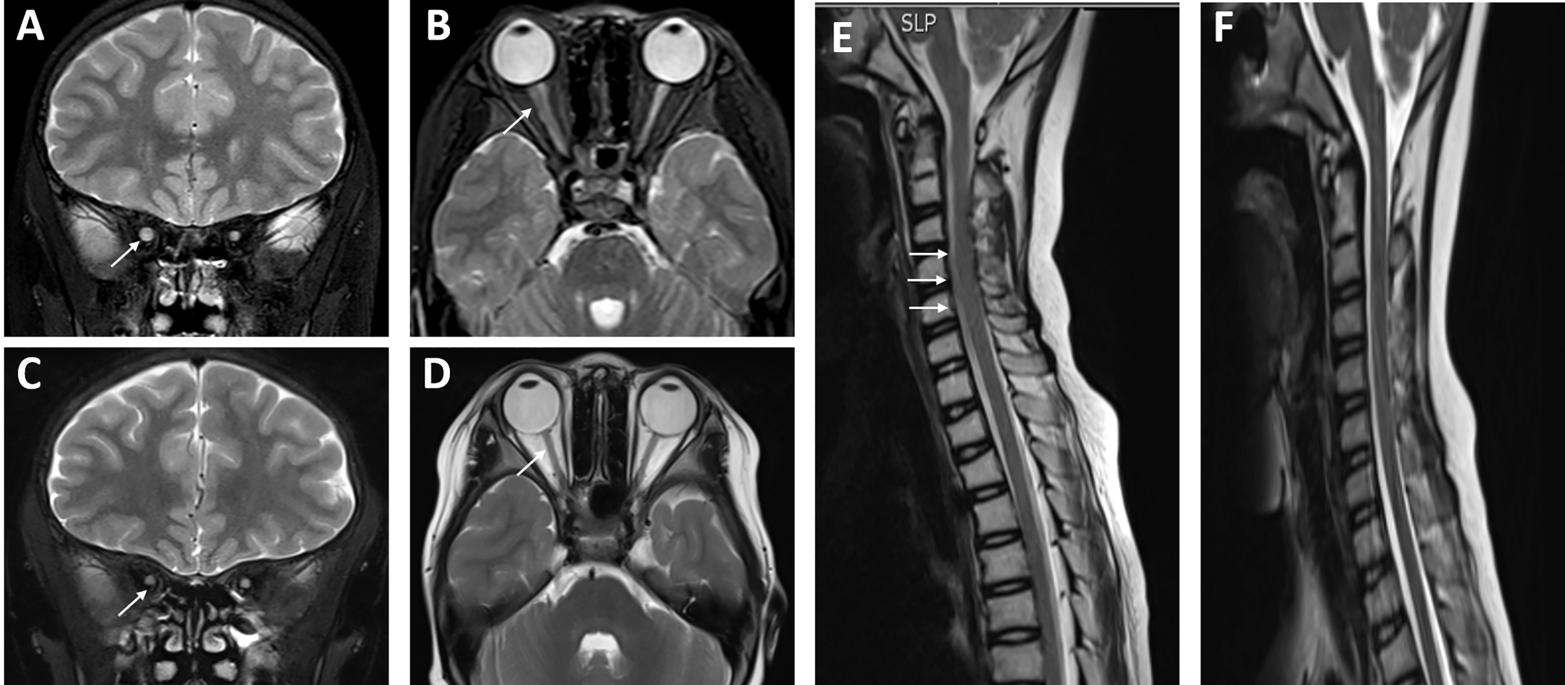
Neuroimaging changes of the patient with systemic lupus erythematosus (SLE)/Sjogren’s syndrome (SS) and NMOSD. **(A, B)** The left optic nerve was obviously smaller than the right through MRI, and there was slight swelling of the spinal cord at the levels of c2-6 **(E)** in December 2018 (shown by arrows). After a powerful immunosuppressive therapy, the left optic nerve was roughly the same size as the right optic nerve in October 2019 **(C, D)**, and the swelling of c2-6 was much better than before **(F)**.

On October 24, 2018, the child had a sudden progressive decline in vision, with dry eyes, lack of tears when crying, but no eye pain. Physical examination showed that the fingers could be identified at 30 cm from the left eye, and the right eye could only have light perception. Two enlarged lymph nodes, about 3 cm in diameter, were palpable in the neck. Coarse breathing sounds and a few moist rales could be heard in both lungs. The liver was impalpable below the costal margin, and the A–B line of the spleen was 5 cm. Nervous psychological reflection of both knees and Achilles tendons showed normal results. No pathological reflection of Babinski’s sign was induced. AQP4-IgG in CSF and serum were with titers of 1:10 and 1:3,200, respectively. MOG-IgG and MBP-IgG in CSF and serum were both negative. Lung CT scan showed interstitial lesions and infections in both lungs (**Figure 2**), while whole exome sequencing (WES) did not detect pathogenic mutations that correlated with clinical manifestations. Additionally, MRI showed no abnormalities in the brain, while the left optic nerve was significantly thinner than the right (**Figures 3A, B**). Furthermore, slight swelling of the cervical spinal cord at c2-6 vertebral levels was detected (**Figure 3E**). The ophthalmologic examination showed that the right eye reacted slowly to light, and only had light perception. Pale optic papillae were found in the left eye through fundus testing, and the relative afferent pupillary defect (RAPD) was positive. Visual evoked potential (VEP) suggested demyelination of bilateral visual pathways mixed with axonal damage. Then, she received high-dose intravenous methylprednisolone for 3 days followed by oral prednisone, gamma globulin, and rituximab (a total of 3 times) to suppress the immune reaction again. Fortunately, the patient’s vision gradually recovered, but her parents refused to reexamine the AQP4-IgG level. After discharge, the child was still treated with prednisone, mycophenolate mofetil, and tacrolimus until now, but recurrent respiratory tract infections appeared. In October 2019, she received continuous positive airway pressure (CPAP) to assist her breathing due to respiratory failure. Fortunately, the left optic nerve was roughly the same size as the right optic nerve in October 2019 (**Figures 3C, D**), and the swelling was much better than before (**Figure 3F**).

## Discussion

In the present study, we reported a case of a girl with onset at age 5, clinically featured by recurrent parotid gland enlargement, pancytopenia, abnormal liver function, hypocomplementemia, multiple positive serum antibodies, and cirrhosis. The disease was initially diagnosed as SS/SLE overlap syndrome. Four years later, the patient had a sudden vision loss, positive AQP4-IgG in both serum and CSF, and involvement of long segmental spinal cord and nervus opticus, consistent with NMOSD diagnosis. So far, this case is the youngest-onset patient with SS/SLE overlap syndrome complicated with NMOSD of Chinese origin, and cirrhosis is a new complication that has never been reported before. At present, the patient is under active preparation for a bone marrow transplant because of the limited response to immune-suppressant treatment.

Both SLE and SS present special challenges in the pediatric population because they are rare and often associated with more severe mortality and worse prognosis compared with adults (12). Onset in childhood shows a higher risk of nephritis, malar rash, anti-dsDNA antibodies, and hemolytic anemia compared with adults with SLE (12), but these symptoms were not detected in our patient. Additionally, recurrent parotid swelling is the most common hallmark of SS, usually proceeding to sicca symptoms in childhood as in our patient, but the incidence is significantly lower than that of adults, which is associated with the age of onset, relatively short course of disease, and the minor damage of exocrine glands such as the salivary gland and lacrimal gland in juveniles (13). Diagnostic criteria for jSS are similar to those proposed in adults, but it manifested a lower sensitivity, and gland biopsy findings of the minor SG criteria (>1 focus of 50 lymphocytes/4 mm^2^) are usually negative in children (14). The autoantibodies associated with jSS, anti-SSA and anti-SSB, are often detectable before overt glandular dysfunction is detected, both positive in the current patient. The continuous positive serum anti-SSA/Ro antibody in the patient might be a high-risk factor affecting the prognosis of the disease, and may be the pathogenic factor responsible for her cirrhosis. The positive rate of anti-SSA and anti-SSB antibodies in children is higher than that in adults; nearly 3/4 jSS patients have higher levels of ESR and IgG than adults, which may be related to the excessive activation of B cells in jSS patients (15). The differences between adults and children in SLE and SS are summarized in **Table 2**.

**Table 2:** Differences between paediatric and adult patients with systemic lupus erythematosus, Sjogren’s syndrome and NMOSD.

Features of the Diseases	SLE		SS		NMOSD	
	Juvenile	Adult	Juvenile	Adult	Juvenile	Adult
Incidence	9.73/100,000	20-150/100,000	Not known	0.01%-0.09%	Not known	0.37-10/100,000
Median age at diagnosis	13 years	37-50 years (female)	10 years	/	11.5 years	32-41 years
Female/male ratio	4:3 before age 10 years	7-15:1	Similar to jSLE	15:01	3:01	9:1
Fatigue, malaise	+++	+++	+++	+++	/	/
Rash	++++	++++	+	++	/	/
Lymphadenopathy	++	++	++	+++	/	/
Raynaud phenomenon	+++	+++	+	++	/	/
Arthralgia/arthritis	+++	+++	+	++	/	/
Oral ulcers	+++	+++	+/-	+/-	/	/
Myalgia/myositis	++	++	+	++	/	/
Cytopenias	+++	+++	+	+	/	/
Neurologic disease	+++	+++	+/-	+++	++	++
Glomerulonephritis	+++	++	+	++	/	/
Glandular enlargement	-	-	+++	++++	/	/
Dry mouth + dry eyes	++	++	+++	++++	/	/
Pulmonary disease	++	++	+/-	++	/	/
Psychonosema	++	++	+/-	++	++	++
Monophasic LETM	/	/	/	/	+/-	++
Recurrent cerebral manifestations	/	/	/	/	++	+
Polyfocal demyelinating	/	/	/	/	++	++
Ophthalmodynia	/	/	/	/	+	+++
Abnormal initial brain MRI	/	/	/	/	++	+
Isolated brainstem syndrome	/	/	/	/	++	+
**Autoantibodies (High vs Low Titers)**						
ANA	++++	++++	+++	++	/	/
Anti-dsDNA	+++	++	-	-	/	/
Anti-RNP	+/- (early)	+/- (early)	-	-	/	/
Anti-Sm	+++	+++	-	-	/	/
Anti-SSA	++	++	+++	+++	/	/
Anti-SSB	++	++	+++	+++	/	/
Low C3 levels	+++	+++	+	+	/	/
Low C4 levels	++	++	+	+	/	/
Anti-AQP4	/	/	/	/	+	+++
Major morbidity and complications	Kidney disease, central nervous system damage, rash, arthritis, oral ulcers, antiphospholipid syndrome, infection, early atherosclerosis	Kidney disease, central nervous system damage, rash, arthritis, oral ulcers, antiphospholipid syndrome, infection, early atherosclerosis; pulmonary fibrosis; neurologic disease	Lymphoma and neurologic disease are rare complications in childhood, corneal abrasions, dental caries, liver function impairment	The incidence of lymphoma is 5%-10%, corneal abrasions, dental caries, renal tubular acidosis, liver function impairment, neurologic disease, pulmonary disease	Monophasic LETM is rare in children, acute disseminated encephalomyelitis, optic neuritis, area postrema syndrome, polyfocal demyelinating clinical, recurrent cerebral manifestations	LETM, acute disseminated encephalomyelitis, optic neuritis, area postrema syndrome
Prognosis	Worse than adults	Mortality is 3 times higer than nomal population	Worse than adults	Increased mortality	High risk for poor recovery	Long-term disability and mortality rates are high

NMOSD is a severe CNS demyelinating disease associated with potential cumulative disability (16). Most clinical features, laboratory characteristics, and neuroimaging of pediatric NMOSD are similar to those of adult-onset disease (17), but the female preponderance may be of lower magnitude (**Table 2**), and a greater proportion of children cases may have the monophasic disease. AQP4-IgG, associated with high disability in both adult (18) and pediatric NMOSD, is detected in the majority of adult patients, but less frequently in children, with a seroprevalence of only 0.7%–4.5% (19). Notably, AQP4-IgG is rarely found in children with monophasic LETM. Childhood-onset ADEM requires a polyfocal demyelinating clinical manifestation with encephalopathy; the presence of AQP4-IgG favors the diagnosis of NMOSD (20). Based on clinical manifestations, neuroimaging, and antibody testing, the NMOSD diagnosis of the current patient was clear. Notably, the left optic nerve was significantly thinner than the right, and then returned to bilateral symmetry after 1 year of powerful immunosuppressive therapy, with improved vision, inconsistent with optic atrophy after SS but consistent with ON in the patient.

Emerging evidence demonstrated that NMOSD often coexists with multiple CTDs (such as SLE, SS, and myasthenia gravis), which are associated with a broad repertoire of autoantibodies. Anti-SSA or anti-SSB antibodies were reported in 7.7%–12% of NMOSD patients, and spinal cord lesions were observed in about 20%–34% of all SS patients (21). SLE with NMOSD is usually presenting with LETM and ON. Previous studies have shown that positive serum AQP4-IgG is rarely found in SS and SLE patients without neurological dysfunction. Literature review is summarized in **Table 3** (22–35). To date, there are few reports about cases in children of SLE coexisting with NMOSD. In 2017, Bushra et al. (26) reported a 15-year-old African American girl who presented with bulbar dysfunction (difficulties in swallowing, speech, and mastication) at age 8, then gait abnormalities, astasia, and distal limb paresthesias appeared 5 months later. Serum AQP-4 antibodies were negative on multiple monitoring and then turned positive until 5 years after the onset. During the process of treatment, the patient manifested obvious mental and behavioral abnormalities and subcutaneous nodules, with multiple positive antibodies (anti-ANA, anti-ENA, and anti-SSA antibodies) in serum. Unfortunately, the disease progressed quickly in the later stage and the outcome was poor. Similar to our patient, Metha et al. (25) reported a woman who developed a 20-year history of SS presenting with myelitis and MRI-diagnosed intracranial lesions. NMO-IgG serology confirmed the diagnosis of SLE/NMOSD, and follow-up found recurrences in subsequent years. However, this case was adult-onset. Several mechanisms for the coassociation of AQP4-IgG and SLE and/or SS have been proposed, such as common genetic or environmental factors. Up to now, 200 genetic loci have been associated with autoimmune diseases (36). Nevertheless, the environmental factors that could be involved in NMOSD are not well described, which makes it harder to investigate these genetic associations. AQP4 is expressed at low levels in salivary glands, whereas AQP5 is expressed at high levels in the salivary gland; there is an approximately 50% protein sequence identity between them (27). The immune system may attack this common sequence in both the CNS and salivary glands, resulting in the coexistence of SS and NMOSD. In addition, AQP4-IgG1 antibodies have been proven to present in patients’ serum several years before the first NMOSD attack with SLE, and the elevation of anti-AQP4 might be a part of a polyclonal B-cell response during NMOSD relapses; Th1 responses also accompany autoantibody responses in the process of NMOSD/SLE (24).

**Table 3:** Literature review.

Diseases	Literature	Information contents
**NMOSD coexisting with SLE**	Birnbaum et al (22)	Two NMOSD patients with a prior history of SLE, one of whom had several relapses after the first episode of NMOSD, the other patient had concurrent anticardiolipin syndrome and cervical myelitis.
	Motaghi et al. (23)	A 34-year-old woman with acute myelitis as the initial symptom and MRI findings typical of NMOSD. Anti-ANA, anti-dsDNA, and anti-cardiolipin antibodies were all positive, also with hypocomple-mentemia. Regrettably, the NMO-IgG detection was unavailable at that time. During the follow-up period, the patient developed typical clinical manifestations of SLE (rash and light sensitivity).
	Piga et al. (24)	104 patients with SLE coexisting with demyelinating syndrome (DS), including 14 patients classified as NMO and 49 patients classified as NMOSD, the majority of whom were women. In 41 patients, DS was the SLE onset manifestation, and LETM was the most frequent manifestation present in 73 patients.
	Kovacs et al. (25)	AQP4-IgG had already existed in sera of 6 SLE patients, and the clinical manifestations of NMOSD appeared in subsequent years.
	Bushra et al. (26)	A 15-year-old African American girl who presented with bulbar dysfunction (difficulties in swallowing, speech, and mastication) at age 8, then gait abnormalities, astasia, and distal limb paresthesias appeared 5 months later. Serum AQP-4 antibodies were negative on multiple monitoring and then turned positive until 5 years after the onset. During the process of treatment, the patient manifested obvious mental and behavioral abnormalities and subcutaneous nodules, with multiple positive antibodies (anti-ANA, anti-ENA and anti-SSA antibodies) in serum.
**NMOSD coexisting with SS**	Javed et al. (27)	25 patients with NMOSD in which 13 out of 24 patients were NMO-IgG-positive and 4 out of 25 patients were diagnosed with SS.
	Kim et al. (28)	Of the 20 patients with labial gland biopsy, 16 were positive, while only 4 patients had elevated anti-SSA antibodiy, emphasizing the important role of labial gland biopsy in early diagnosis of SS. Subsequently, identified 8 patients were identified with spinal cord involvement from 112 patients with SS referred to the neurology department, and 7 of them met the diagnostic criteria for NMO and with positive AQP4-IgG
	Qiao et al. (29)	616 patients with SS at Peking Union Medical College Hospital, 43 of whom developed NMOSD, with an incidence rate of 7.0%. Moreover, LETM was found in 2 of 22 SS patients, of which only 1 was positive for AQP4-IgG, while no AQP4-IgG was detected in the other 21 patients, including patients with short transverse myelitis (<3 vertebral segments).
	Kolfenbach et al. (30)	17 patients with acute myelitis for 12 years who were diagnosed with SS (6), SLE (5), SS/SLE overlap (2), MS/SS overlap (2), and NMO (2). NMO-IgG was found positive in 4 SS patients, but was not detected in SLE patients. 8 CTD patients met the diagnostic criteria for NMOSD, and 6 NMO-IgG-positive patients relapsed, compared with only three NMO-IgG-negative patients.
	Akaishi et al. (31)	4,447 patients suspected of having NMOSD with acute neurological episodes and the presence of SS-related symptoms, and 1,651 were positive for serum AQP4-IgG. Compared to AQP4-IgG-negative patients, the prevalence of SS was much higher among AQP4-IgG-positive. Additionally, comorbid SS was more prevalent in females, and it had a higher relapse frequency among AQP4-IgG-positive patients.
	Min et al. (32)	A case in 2010 in which the patient developed SS and intracranial lesions at the early stage, and NMO-IgG was tested positive one year later. The patient relapsed several times in the following years, but never had ON or LETM.
**NMOSD coexisting with SLE or SS**	Yasuhiro et al. (33)	626 hospitalized patients with SLE or SS were retrospectively studied, and sera from 6 patients with suspected NMOSDs and SLE (3) or SS (3) were evaluated. As a result, 2 patients’ (1 with SLE and 1 with SS) sera samples were positive for AQP4-IgG.
	Martín-Nares et al. (34)	A study included 12 patients with the diagnosis of NMOSD, of whom 7 had SLE and 5 SS. In 5 patients NMOSD followed SLE/SS onset, 4 had a simultaneous presentation, and in 3 NMOSD preceded SS onset.
**NMOSD coexisting with SS/SLE**	Metha et al. (35)	A woman who developed with a 20-year history of SS presenting with myelitis and MRI-diagnosed intracranial lesions. NMO-IgG serology confirmed the diagnosis of SLE/NMOSD, and follow-up found recurrences in subsequent years.

At present, there are no treatment guidelines and specific recommendations for the treatment of SS/SLE overlap syndrome with NMOSD worldwide. In the acute stage of SLE complicated with NMOSD, high-dose intravenous (IV) steroids followed by plasma exchange (PLEX) could efficiently remove pathogenic antibodies and complements in serum. If patients had minimal response to steroids and PLEX, IV immunoglobulin or cyclophosphamide and high-dose methotrexate (MTX) could be considered (2). In 2010, the European Federation of Neurological Societies (EFNS) has recommended cyclophosphamide as second-line therapy for NMOSD associated with CTDs such as SLE and SS (35). For the main long-term immunosuppressant therapies, rituximab, mycophenolate mofetil, and azathioprine act as effective preventive therapeutic strategies. Notably, eculizumab, a humanized monoclonal antibody, is the first FDA-approved medication in 2019 to be used for chronic treatment of seropositive NMOSD by inhibiting the terminal complement protein C5 (36). Moreover, a phase I study of eculizumab in patients with SLE showed successful complement inhibition with only mild adverse effects (37). Tocilizumab, an L-6 receptor antagonist, is also considered a therapeutic option for SS/NMOSD patients with poor response to rituximab (38). Although pediatric patients had more severe mortality and worse prognosis compared with adults, the combination of anti-humoral and anti-cellular immunity should be considered for specific patients with both NMOSD and CTDs (39). However, infliximab, adalimumab, or etanercept should potentially be avoided because of the exacerbation of CNS demyelinating disease.

## Conclusion

This study reported the first case of preschool-onset SS/SLE overlap syndrome with NMOSD of Chinese origin, and cirrhosis was also present on this basis. Additionally, considering the possibility of autoimmune hepatitis, the age scope and clinical phenotype of CTDs complicated with NMOSD were further expanded. In future clinical work, for SS/SLE children with young-onset age, it is recommended to perform labial and salivary gland biopsies as early as possible to improve the positive rate of diagnosis, regularly monitor relevant antibodies in the blood, track whether central nervous system symptoms occur during the course of the disease, and test NMO-IgG as early as possible to avoid missed diagnosis. Once CTDs or CTDs coexisting with NMOSD are diagnosed, the treatment of immunosuppressants should be actively carried out to improve the prognosis and quality of life of patients.

## Data Availability Statement

The original contributions presented in the study are included in the article/supplementary material. Further inquiries can be directed to the corresponding author.

## Ethics Statement

The studies involving human participants were reviewed and approved by the Animal Ethics Committee, the Second Xiangya Hospital of Central South University, China. Written informed consent to participate in this study was provided by the participants’ legal guardian/next of kin. Written informed consent was obtained from the individual(s), and minor(s)’ legal guardian/next of kin, for the publication of any potentially identifiable images or data included in this article.

## Author Contributions

Conception and design of the study: LJL, LQL, and XL. Performance of experiments: LJL and YX. Data analysis: LJL and PH. Contributed reagents/materials/analysis tools: LQL and LT. Paper writing: LJL, JX, and LZ. All authors contributed to editorial changes in the manuscript. All authors read and approved the final manuscript.

## Funding

This project was supported by China National Natural Scientific Foundation grants (Nos. 81873762 and 81501039), Science and Technology Department of Hunan Province Funds (Nos. 2022SK2032 and 2018SK2069), and Health Select Commission of Hunan Province Funds (No. B20180311).

## Conflict of Interest

The authors declare that the research was conducted in the absence of any commercial or financial relationships that could be construed as a potential conflict of interest.

## Publisher’s Note

All claims expressed in this article are solely those of the authors and do not necessarily represent those of their affiliated organizations, or those of the publisher, the editors and the reviewers. Any product that may be evaluated in this article, or claim that may be made by its manufacturer, is not guaranteed or endorsed by the publisher.

## Abbreviations

SLE, systemic lupus erythematosus; CTD, connective tissue disease; SS, Sjogren’s syndrome; NMOSD, neuromyelitis optica spectrum disorder; CNS, central nervous system; CSF, cerebrospinal fluid; ON, optic neuritis; LETM, longitudinally extensive transverse myelitis; APS, area postrema syndrome; BBB, blood–brain barrier; MS, multiple sclerosis; ESR, erythrocyte sedimentation rate; ECG, electrocardiogram; WES, whole exome sequencing; RAPD, relative afferent pupillary defect; VEP, visual evoked potential; CPAP, continuous positive airway pressure; DS, demyelinating syndrome; IV, intravenous; PLEX, plasma exchange; MTX, methotrexate; EFNS, European Federation of Neurological Societies.
